# Long-term survival after pancreatic metastasis resection from breast cancer: a systematic literature review

**DOI:** 10.1186/s40792-021-01124-8

**Published:** 2021-02-03

**Authors:** Atsuki Nagao, Tamaki Noie, Hajime Horiuch, Haruyasu Yamada, Masashi Momiyama, Kentaro Nakajima, Shouichi Satou, Hitoshi Satodate, Satoshi Nara, Yasushi Harihara

**Affiliations:** 1 Department of Surgery, NTT Medical Center Tokyo, 5-9-22, Higashi-Gotanda, Shinagawa-ku, Tokyo, 141-8625 Japan; 2 Department of Pathology, NTT Medical Center Tokyo, Tokyo, Japan; 3 Department of Radiology, NTT Medical Center Tokyo, Tokyo, Japan

**Keywords:** Breast cancer, Pancreatic metastasis, Pancreatectomy, Oligometastases

## Abstract

**Background:**

Patients with advanced-stage breast cancer often demonstrate pancreatic metastases. However, pancreatic metastases resection from breast cancer has been rarely performed, with only 20 cases having been reported to date.

**Case presentation:**

A 49-year-old woman presented to our hospital in September 2003 with complaints of uncontrollable oozing from her left breast tumor. Computed tomography revealed a left breast tumor approximately 9.3 cm in diameter as well as heterogeneously enhanced solid mass lesions with necrotic foci in the pancreatic tail and body, up to 6.2 cm, which were radiologically diagnosed as pancreatic metastases from breast cancer. An emergent left simple mastectomy was performed to control bleeding. After epirubicin and cyclophosphamide hydrate treatment failed to improve her condition, the pancreatic metastases responded to weekly paclitaxel treatment, but eventually regrew. The patient underwent distal pancreatectomy with splenectomy, left adrenalectomy, partial stomach resection, and paraaortic lymph nodes excision in December 2004 after no other metastasis was confirmed. Furthermore, she received radiation therapy for left parasternal lymph node metastasis 6 months later. The patient recovered well. Consequently, she has no evidence of disease > 15 years after pancreatectomy.

**Conclusions:**

This is the first reported case of pancreatectomy for pancreatic metastases from breast cancer, which was simultaneously diagnosed. Patients with no metastasis other than resectable pancreatic metastases and breast cancer and who possess some sensitivity for chemotherapy may benefit from pancreatectomy.

## Background

A large autopsy series found that the prevalence of pancreatic metastasis is as high as 11%. Lung, colon, and breast cancers are the most common tumors of origin [1]. However, pancreatic metastasis resection from breast cancer is extremely rare with only 20 cases reported [2–18]. This paper presents a case of a patient simultaneously diagnosed with breast and pancreatic cancer with no evidence of disease 15 years after undergoing distal pancreatectomy (DP).

## Case presentation

**Fig. 1:**
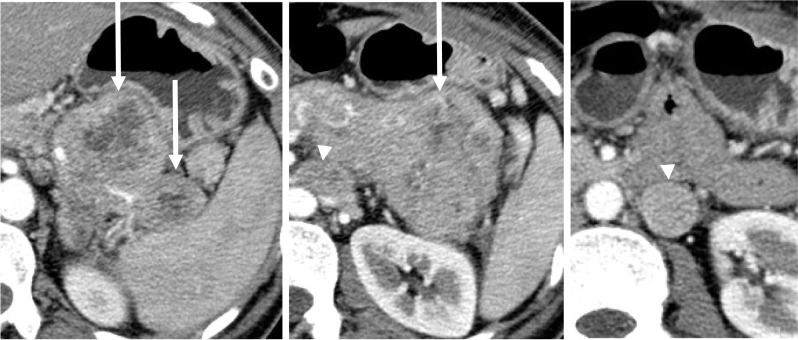
Pancreatic metastases and paraaortic lymph node metastases. Late arterial phase of dynamic contrast-enhanced computed tomography showing heterogeneously enhanced multiple solid mass lesions with necrotic foci in the pancreatic tail and body up to 6.2 cm (arrow) with peripancreatic and paraaortic lymph nodes swelling (arrowhead)

**Fig. 2:**
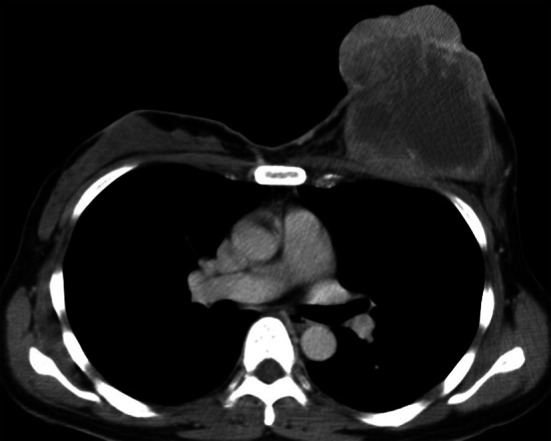
Left breast cancer. Contrast-enhanced computed tomography showing the tumor projecting to the skin, approximately 9.3 cm in diameter

**Fig. 3:**
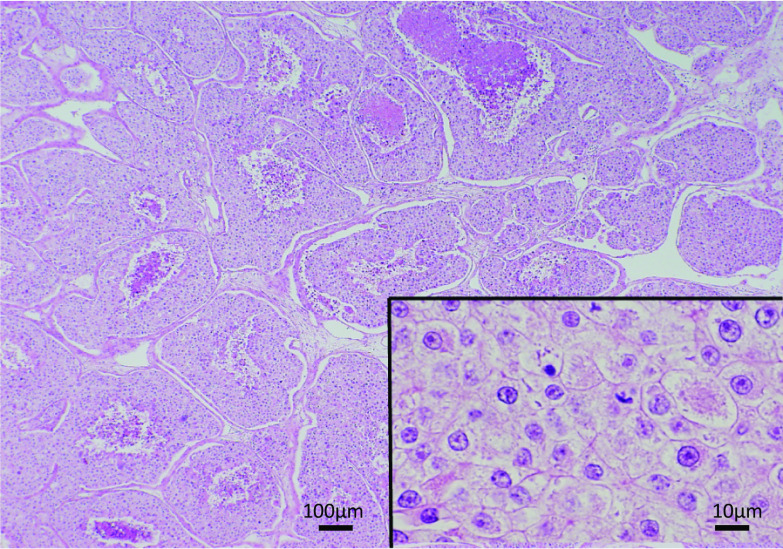
Histology of left breast cancer. The tumor is composed of large nests with central comedo necrosis. The tumor cells have large round vesicular nuclei, prominent nucleoli, and pale eosinophilic granular cytoplasm

A 49-year-old woman complaining of uncontrollable oozing from her left breast tumor presented to our hospital in September 2003. She had been aware of her left breast tumor for > 10 years and had self-managed her left breast erosion for > 3 years. Laboratory test results revealed anemia (hemoglobin 9.1 g/dL) and elevated levels of carcinoembryonic antigen (CEA; 65.2 ng/mL) and carcinoma antigen 15-3 (44.2 U/mL). However, the carbohydrate antigen 19-9 value was not elevated (12.1 U/mL). Furthermore, the late arterial phase of dynamic contrast-enhanced computed tomography (CT) revealed heterogeneously enhanced multiple solid mass lesions with necrotic foci in the pancreatic tail and body [19]—up to 6.2 cm in size—with peripancreatic and paraaortic lymph node swelling (Fig. 1). A left breast tumor projecting to the skin approximately 9.3 cm in diameter (Fig. 2) as well as left axillary lymph node swelling was simultaneously noted. The largest pancreatic mass, in this case, was a solid mass that was contrast-enhanced to the same extent as the surrounding pancreatic parenchyma although the common imaging finding of pancreatic ductal carcinoma is hypovascular. This mass was also not hypervascular enough to raise the suspicion of a pancreatic neuroendocrine tumor. In addition, other noncontiguous pancreatic masses were found, and the presence of breast cancer was suggested, which led to the suspicion of pancreatic metastases from breast cancer rather than the primary pancreatic tumor, radiologically. The next day, emergent left simple mastectomy and reconstruction of the left breast by inferior lateral rectus abdominis muscle flap [20] were performed to control bleeding. Macroscopically, the tumor was 7 cm in diameter and protruded from the skin. The cut surface of the tumor was solid and grayish-white with central necrosis, and the surgical margin was negative. Microscopically, the tumor was composed of large nests with central comedo necrosis. The tumor cells had large, round vesicular nuclei, prominent nucleoli, and pale eosinophilic granular cytoplasm. Mitotic figures were frequently observed (Fig. 3). Immunohistochemically, the tumor was negative for estrogen receptor (ER) and progesterone receptor (PR), and the HercepTest score was 0.

She began chemotherapy 2 weeks later with epirubicin and cyclophosphamide hydrate once every 3 weeks. Her CEA level increased (83.6 ng/mL) after four treatment cycles, and CT demonstrated a slight expansion of the pancreatic tumor. Weekly paclitaxel treatment started in December 2003. Her CEA level decreased gradually (to a minimum of 6.1 ng/mL in June 2004), and CT performed in July 2004 revealed shrunken pancreatic tumors that could not be recognized as masses with different densities. Thereafter, her CEA level increased again (up to 14.0 ng/mL in December 2004), and the pancreatic tumor regrew. Recurrent peripancreatic and paraaortic lymph node swelling was noted on CT performed in October and December 2004. Bone scintigraphy showed no evidence of bone metastasis, and CT revealed left axillary lymph node swelling to keep vanishing.

**Fig. 4:**
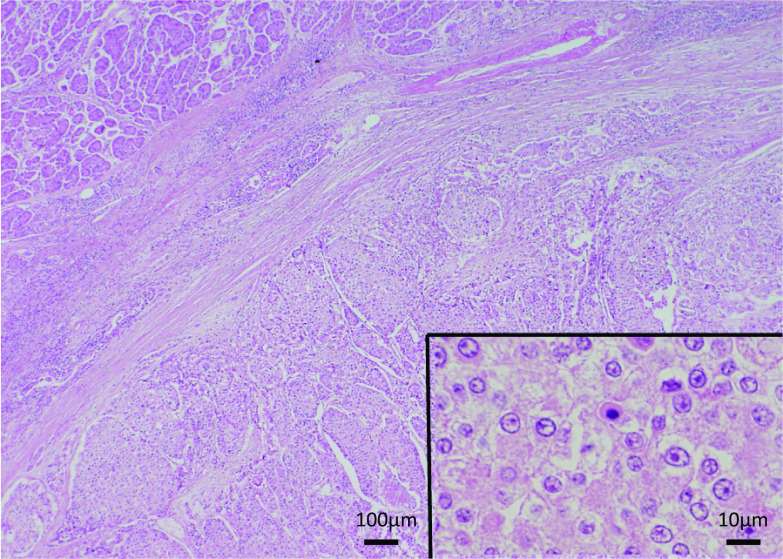
Histology of the metastatic pancreatic tumor. The tumor, composed of large nests, invades the pancreas. The tumor cells have large round vesicular nuclei, prominent nucleoli, and pale eosinophilic granular cytoplasm

She underwent DP with splenectomy, left adrenalectomy, partial stomach resection, and paraaortic lymph nodes excision in December 2004. A gross examination of the resected specimen revealed a multinodular tumor at the pancreas tail, 3.5 × 3 × 2 cm in size. The tumor invaded the lymph nodes around the pancreas. The cut surface of the tumor was solid and grayish-white with focal hemorrhaging. Microscopically, the tumor invaded the pancreas and surrounding tissue, forming solid nests with central necrosis. The tumor cells had large round vesicular nuclei with prominent nucleoli and pale eosinophilic granular cytoplasm. Mitotic figures were frequently found (Fig. 4). These histological features were similar to those of this patient’s primary breast cancer (Fig. 3). Immunohistochemically, the tumor was negative for ER, PR, gross cystic disease fluid protein-15, and mammaglobin. Although immunohistochemical markers of breast cancer were negative, the patient was diagnosed with primary breast cancer metastases to the pancreas based on the histological similarity between these two tumors.

Ultrasonography and CT performed in May 2005 revealed left parasternal lymph node metastasis, which was subsequently treated by radiation therapy comprising 60 Gy/30 f. She took 5′-deoxy-5′-fluorouridine for 2 years after pancreatectomy. The patient was doing well with no evidence of disease as of January 2020.

## Discussion and conclusions

Metastatic pancreatic tumor resection has been increasingly reported in proportion to decreased morbidity and mortality after pancreatectomy [21–23]. Careful patient selection can improve the survival of patients with metastatic pancreatic cancer after undergoing pancreatectomy [23].

In contrast, few reports exist of pancreatic metastasis resection from breast cancer [2–18], which are not rare in those with advanced-stage disease [1, 24]. The long-term prognosis after pancreatic metastasis resection from breast cancer is unclear. The present patient has survived > 15 years after undergoing pancreatectomy. Herein, other reported patients with breast cancer who underwent pancreatic metastases resection were reviewed.

Only 20 breast cancer patients who underwent resection of pancreatic metastases (Table 1) have been reported [2–18]. However, only two patients were simultaneously diagnosed with pancreatic and breast cancer. They underwent simultaneous pancreaticoduodenectomy (PD) and mastectomy after being diagnosed with papilla of Vater and pancreatic head cancers, respectively [12, 14]. In the remaining 18 cases, pancreatic tumors were diagnosed metachronously with intervals ranging between 2 months and 26 years after breast cancer resection. Moreover, no reported case underwent neoadjuvant chemotherapy before pancreatectomy.

**Table 1:** Reported cases undergoing pancreatic metastasis resection from breast cancer

Case no.	Ref.^a^	Age^b^ (years)	Timing^c^	Interval^d^ (months)	Preop diag^e^	Op^f^	LN^g^	Outcome	Duration (months)
1	2	52	Meta^h^	38	Panc^i^	PD^j^		Alive	72
2	3	33	Meta^h^	30	Panc^i^	PD^j^	−	Alive	27
3	4	46	Meta^h^	80	Papilla^k^	PD^j^		Alive	12
4	5	70	Meta^h^	36	Panc T^l^	PpPD^m^		Alive	37
5	5	46	Meta^h^	60	Panc T^l^	PpPD^m^		Alive^n^	21
6	5	57	Meta^h^	84	Panc T^l^	PpPD^m^		Dead	26
7	6	75	Meta^h^	312	Panc T^l^	Enucleation	−	Alive	80
8	7	57	Meta^h^	54	Breast meta^o^	DP^p,q^	+ ^r^	Alive^n^	5
9	8	60	Meta^h^	2?	Bile duct^s^	PD^j^		Alive^n^	2
10	8	55	Meta^h^	114	Panc tail T^t^	DP^p^	+ ^r^	Alive	2
11	9	41	Meta^h^	60	Breast meta^o^	DP^p^		Alive	
12	10	53	Meta^h^	20	Panc^i^	PD^j^	+	Dead	36
13	11	75	Meta^h^	216		PD^j^	+	Alive^n^	48
14	12	68	Simul^u^	0	Papilla^k^	PD^j^	−	Alive	12
15	13	34	Meta^h^	19		PD^j^		Alive	12
16	14	48	Simul^u^	0	Panc^i^	PD^j^	−	Dead	12
17	15					PD^j^			
18	16	35	Meta^h^	45		TP		Dead	7
19	17		Meta^h^				−	Dead	13
20	18	47	Meta^h^	41		PD^j^		Dead	28
Present case		49	Simul^u^	0	Breast meta^o^	DP^p^	+ ^r^	Alive	180

Each case from case nos. 15 to 20 is one among many cases in each report, such as the cases with resection of pancreatic metastasis

^a^Reference number

^b^Age at diagnosis of pancreatic tumor

^c^Timing of the diagnosis of breast cancer and pancreas tumor, simultaneous or metachronous

^d^Interval between breast cancer surgery and diagnosis of pancreatic tumor

^e^Preoperative or intraoperative diagnosis of pancreatic tumor

^f^Operative procedure

^g^Lymph node metastasis from pancreatic metastasis

^h^Metachronous

^i^Pancreatic head cancer

^j^Pancreaticoduodenectomy

^k^Cancer of the papilla of Vater

^l^Pancreatic head tumor

^m^Pylorus-preserving pancreaticoduodenectomy

^n^Alive with recurrence

^o^Metastatic pancreatic tumor from breast cancer

^p^Distal pancreatectomy

^q^Distal pancreatectomy with concomitant resection of the left kidney, left adrenal gland, and partial colon

^r^Paraaortic lymph node metastasis

^s^Bile duct cancer

^t^Pancreatic tail tumor (adenocarcinoma was confirmed by frozen section)

^u^Simultaneous

Preoperative diagnosis of pancreatic metastasis was achieved in only two cases. One was diagnosed with pancreatic metastasis from breast cancer by radiologic examination [7], as in the present case, and another was diagnosed by fine-needle aspiration cytology under endoscopic ultrasonography [9]. In the remaining 18 patients, the preoperative diagnosis was pancreatic head cancer or another one other than pancreatic metastasis from breast cancer. The final diagnosis was achieved after a pathological examination. PD (including pylorus-preserving PD), DP, total pancreatectomy, and enucleation were performed in 14, 4 (including the present case), 1, and 1 case, respectively. However, the surgical method was not reported in one case. Lymph node metastases from pancreatic metastases were present in 5 of 10 cases.

Six patients expired between 7 months and 3 years after undergoing pancreatectomy, and 13 cases, including four cases with recurrence, survived from 2 months to 6.7 years. Two patients survived without a recurrence for > 5 years after undergoing pancreatectomy [2, 6]. The cumulative 5-year survival rate is as high as 52%, partly owing to many cases having been reported to be alive.

Metastatic breast cancer (MBC) is generally an incurable disease and its prognosis is unfavorable [25, 26], especially in patients with triple-negative breast cancer (TNBC) [27, 28], defined by negative expressions of ER and PR and the lack of amplification of the human epidermal growth factor-2 (HER2) gene. Systemic therapy is the standard care for patients with MBC [29, 30]. However, selected patients with very limited or oligometastatic distant disease may benefit from the addition of metastases-directed local therapy on rare occasions [31, 32]. Patients with liver metastases from breast cancer, which are more frequently encountered than pancreatic metastases, are indicated for hepatectomy only if they have favorable prognostic markers, such as positive ER, HER2 overexpression, or stable disease after neoadjuvant therapy [33–35]. The study on stereotactic body radiotherapy (SBRT) for patients with oligometastases mentioned that MBC patients who experienced stable or regressive disease after systemic therapy before SBRT survived significantly longer than MBC patients who experienced lesion progression [36]. However, pancreatic metastases were progressive following regression before pancreatectomy in this patient, and her cancer was TNBC. For pancreatic metastasis from breast cancer, the favorable prognostic features after pancreatectomy are uncertain because of the few numbers of cases. Thus, if a patient has no metastasis other than resectable pancreatic metastases and is sensitive to chemotherapy, she may benefit from pancreatectomy because no biomarker for patient identification with the true oligometastatic disease is clinically available^37^.

The patient in this study was the first to be simultaneously diagnosed with pancreatic metastases with breast cancer and undergo pancreatectomy. Moreover, the present case highlights the potential for long-term survival after resection of pancreatic oligometastases from breast cancer.

## Data Availability

The datasets generated and/or analyzed during the current study are not publicly available because individual privacy could be compromised, but are available from the corresponding author on reasonable request.

## Abbreviations

CEA: Carcinoembryonic antigen
CT: Computed tomography
DP: Distal pancreatectomy
ER: Estrogen receptor
HER2: Human epidermal growth factor-2
MBC: Metastatic breast cancer
PD: Pancreaticoduodenectomy
PR: Progesterone receptor
SBRT: Stereotactic body radiotherapy
TNBC: Triple-negative breast cancer
